# Convenient design method for customized implants based on bionic vein structure features

**DOI:** 10.3389/fbioe.2022.929133

**Published:** 2022-08-12

**Authors:** Lin Wang, Weizhong Geng, Kunjin He, Kaijin Guo

**Affiliations:** ^1^ School of Medical Information and Engineering, Xuzhou Medical University, Xuzhou, China; ^2^ College of Computer and Information Engineering, XinXiang University, XinXiang, China; ^3^ College of Internet of Things Engineering, Hohai University, Changzhou, China; ^4^ Department of Orthopedics, Affiliated Hospital of Xuzhou Medical University, Xuzhou, China

**Keywords:** bionic vein structure, feature, orthopedic implants, customized design, digraph structure

## Abstract

Matching implants to bones is crucial for customized orthopedic medicine. Existing methods for designing customized implants predominantly adopt the parameterized deformation method that uses a fragmented representation of semantic parameters. Such a representation cannot provide information integration management and therefore restricts the retrieval of information regarding implant features and the improvement of customized design efficiency. Therefore, this study proposes a rapid design method for customized implants based on bionic vein structure features. First, a bionic vein structure was designed to represent the implant type. Second, the bionic vein structure was represented by a digraph structure with morphological and dimensional features. Finally, the implant model was rapidly built by retrieving the sketch and other modeling operations. Common implants such as the T-shaped plate, L-shaped plate, clover plate, and femoral stem prosthesis were used as explanations or test cases. The experimental work shows that combining the traditional parametric deformation method with bionic vein structure features in our present method is flexible and efficient results, and can improve the efficiency of customized implant design.

## 1 Introduction

Implants can treat musculoskeletal diseases effectively (Kubicek et al., 2019; Farii et al., 2022). With the increase in personalized medical needs, the demand for customized orthopedic implants has risen (Thomas, 2021). The existing design methods hardly achieve the reuse of implant, especially with the complex abutted surfaces. Inappropriate implants can lead to many clinical complications (Harith et al., 2016; Zubairi et al., 2017). Studies show that most musculoskeletal diseases are closely related to the bone local anatomy (Tommas ini et al., 2010), bone size (Sindhu and Soundarapandian, 2019; Frysz et al., 2020; Kulkarni et al., 2020), and fracture type (Chen et al., 2022). Thus, it is particularly important to design customized implants according to the anatomic characteristics of the patient’s native bone (Hwang et al., 2012; Ravindra et al., 2017).

In commercial modeling software (such as AutoCAD, CATIA, and SolidWorks) (Andreas et al., 2018; Soni and Singh, 2020), customized implants are constructed through a series of complex interactive operations on point, line, and surfaces. The disadvantage is that the implant redesign is hardly achievable. So, the parametric design was proposed to meet the free deformation of implant shape and size to designing customized implants. Babaniamansour et al. (2017) improved the toughness of the femoral stem by deploying a novel design using biomechanics and biomaterials. Hongwei et al. (Liu, 2015) designed a customized artificial femoral prosthesis with a better matching degree between the prosthesis and the femoral metaphyseal medullary cavity. Although the above-mentioned studies improved the efficiency of implants’ design, the concept of integration was still never considered. This resulted in repeated communication between orthopedic surgeons and implant designers, which undoubtedly increased the complexity of the entire design process (Liu et al., 2019; Soni and Singh, 2020).

Subsequently, with the development of feature technology, feature idea has been applied to implant design (Camba et al., 2016; Cheng and Ma, 2017; Jabran et al., 2019). For instance, Chen et al. (2021) proposed a computer-aided approach to explore the design and modification of customized plates. (Kunjin et al., 2015; He et al., 2019) proposed a novel approach for designing customized orthopedic plates based on the bone template and plate semantic feature parameters, thus avoiding tedious operations and individually producing full designs for each plate. At the same time, semantic feature parameters were further used to integrate the bone geometric features into the representation of the bone plate features in our previous research (Wang et al., 2017; Wang et al., 2021). More importantly, a hierarchical mapping relationship was established between the bone plate and the bone to improve the matching degree of the bone and plate surfaces. However, using only semantic feature parameters inescapably resulted in information fragmentation, which limited the retrieval of information about the implant features. Regrettably, this problem has so far remained unresolved.

With further study of implant design, we gradually realized that shape features can improve the accuracy and efficiency of retrieval (Pan et al., 2016; Wang et al., 2019). To do this, a novel design method based on bionic vein structure is proposed in this research. First, the adjacency matrix based on the bionic vein structure feature is adopted to express the implant shapes and size. Then, the implant modification and redesign are achieved efficiently by using the adjacent matrix of graph structure based on bionic vein structure. Experimental results showed that this method is simple, flexible, and can greatly improve integration and facilitate feature retrieval and modification.

The rest of this study is organized as follows. In Section 2, the proposed method is presented. Section 3 presents the implementation of the approach and examples used. Finally, Section 4 presents the conclusion of the study and proposes future research directions.

## 2 Materials and methods

**FIGURE 1:**
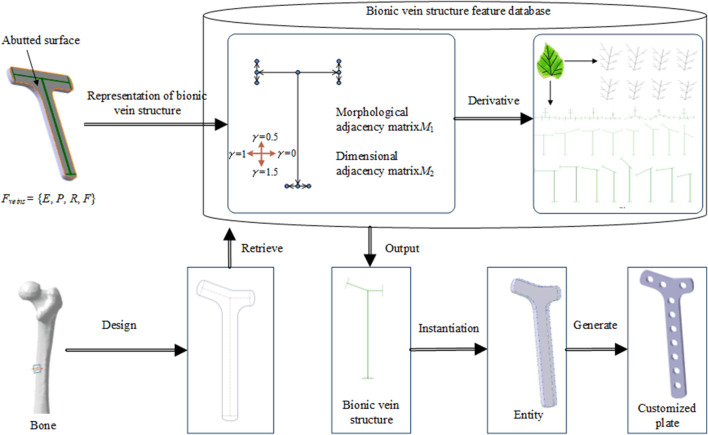
Flowchart of the proposed design method.

Figure 1 shows the proposed method for implants based on the bionic vein structure feature. The steps for this method are as follows:

Step 1: Implant features are represented as a multivariate set that includes geometric elements, semantic parameters, constraint relations, and mapping relations. Among them, the bionic vein structure is simultaneously used to represent the implant's abutted surface.
Step 2: The bionic vein structure is represented by a digraph, and the vertices and weights are set to construct an adjacency matrix with morphological and dimensional information, respectively. The bionic vein structure of different types of implants is represented by a digraph structure, which is stored in the relevant database.
Step 3: Based on bone data and clinician requirements, the implant type and size are determined. The bionic vein structure features with the highest matching degree are first retrieved from the database, and then the required structures are generated based on the parametrization of the bionic vein structure features, and the customized implant is generated through entity construction.

### 2.1 Implant feature representation based on the bionic vein structure

#### 2.1.1 Method description

Parametric methods are usually used to achieve rapid implant design. Although semantic parameters facilitate the size modification of implants, they alone cannot rapidly retrieve implant information. Therefore, in the implant feature representation, the implant abutted surface is first represented by the bionic vein structure (the details are discussed in Section 2.2), and the vein structure contains the vein feature points and curves. Next, high-level semantic parameters are set based on the bionic vein structure. Hence, the representation of implant features facilitates the retrieval and design of the implant.

A formalized representation of implant features as a multiple set *F*_*feature*_ is specifically expressed as follows: *F*_*feature*_ = {*E*_*implant*_, *P*_*implant*_, *R*_*implant*_, *F*_*implant*_}, where *E*_*implant*_ represents geometric elements, *P*_*implant*_ represents semantic parameters, *R*_*implant*_ represents constraint relationships between geometric elements, and *F*_*implant*_ represents a mapping between topological and semantic elements. *F*_*feature*_ is as follows:

a) *E*_*implant*_ = {*E*_p_, *E*_c_, *E*_s_, *E*_e_}.
  1) E_p_ represents the set of feature points, *E*_c_ represents the set of feature curves, *E*_s_ represents the abutted surface, and *E*_e_ represents the entity, Wherein, *E*_p_ represents the collection of feature points, including the main vein feature points *E*_p0_, the lateral vein feature points *E*_p1_, and the minor vein feature points *E*_p2_.
  2) *E*_c_ represents the set of feature curves [*E*_c_ = { *E*_c0_, *E*_c1_ }], wherein, *E*_c0_ includes the main vein feature curves *E*_c00_, the lateral vein feature curves *E*_c01_, and the minor vein feature curves *E*_c02_; *E*_c1_ represents the boundary feature curve, which is generated by connecting the feature points contained in *E*_p_.
  3) *E*_s_ represents the abutted surface, which is constructed by a surface construction method (such as filling) on the basis of *E*_p_ and *E*_c_.
  4) *E*_e_ represents an entity, which is constructed and generated by an entity construction method (such as stretching) on the basis of *E*_s_.
b) *P*_*implant*_ = {*P*_0_, *P*_1_, *P*_2_, *P*_3_, *P*_4_}.

where *P*_0_ represents the main vein parameters, primarily the length of the main vein, *P*_1_ represents the lateral vein parameters, primarily the length of the lateral veins, *P*_2_ represents the minor vein parameters, primarily the length of the minor vein, *P*_3_ represents the direction parameter, primarily the included angle between the veins, and *P*_4_ represents solid parameters such as the thickness of solid and the diameter of the screw hole.

c) *R*_*implant*_ represents the constraint relations between sets *E*_*implant*_ and *P*_*implant*_, primarily the topological relations among points, curves, and surfaces (Wang et al., 2016).
d) *F*_*implant*_ = {*F*_0_, *F*_1_}.

where *F*_0_ represents the two-level mapping relationship (Wang et al., 2016), and *F*_1_ represents the semantic parameter mapping relationship, as follows:

$$F_{0}=\left\{F_{00},\;F_{01}\right\}$$

$$F_{00}=\left\{X_{p}\to Y_{c},\;|X_{p}\in E_{p},Y_{c}\in E_{c}\right\}$$

$$F_{01}=\left\{Y_{c}\to Z_{s},\;|Y_{c}\in E_{c},Z_{s}\in E_{s}\right\}$$

$F_{1}=\left\{H_{y}=f\left(H_{x}\right)|H_{x}\in P,\;H_{y}\in P\right\}$ , where *f* is the relation function.

#### 2.1.2 Example analysis

**FIGURE 2:**
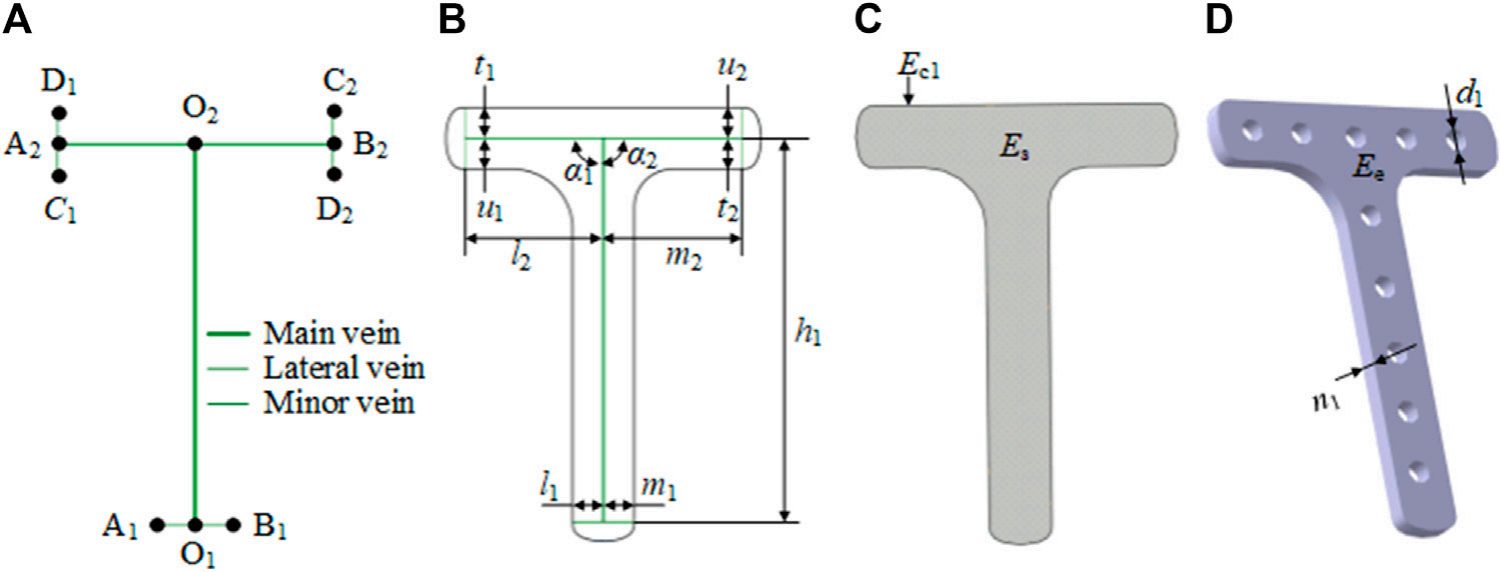
Characteristic representation of the T-shaped bone plate based on the bionic vein structure. **(A)** Vein structure of T-shaped bone plate, contains main vein, lateral vein, minor vein, and vein feature points. The vein feature points include the main vein feature points (O_1_, O_2_), the lateral vein feature points (A_1_, B_1_, A_2_, B_2_), and the minor vein feature points (C_1_, D_1_, C_2_, D_2_). **(B)** Semantic parameters of T-shaped bone plate, mainly refers to the length of veins and the angle between veins. **(C)** Abutted surface of T-shaped bone plate constructed by filling on the basis of panel **(A, B)**. **(D)** Entity of T-shaped bone plate generated by stretching on the basis of panel **(C)**.

In our research, we considered a specific T-shaped bone plate as an example of detailed explanation. For the T-shaped bone plate shown in Figure 2, the center line O_1_O_2_ is selected as the main vein. The lower end of the vein branches out into the left lateral vein O_1_A_1_ and the right lateral vein O_1_B_1_, to define the tail width of the bone plate. The upper-end branches out into the left lateral vein O_2_A_2_ and right lateral vein O_2_B_2_, and O_2_A_2_ continues to branch out into the left minor vein A_2_C_1_ and right minor vein A_2_D_1_, while O_2_B_2_ continues to branch out into the left minor vein B_2_C_2_ and right minor vein B_2_D_2_. These branches are used to define the width head of the bone plate. The feature of the T-shaped bone plate is expressed as *F*_T-shaped_ = {*E*, *P*, *R*, *F*}, where:

a) *E*_p0_ = {O_1_, O_2_}.
b) *E*_p1_ = {A_1_, B_1_, A_2_, B_2_}.
c) *E*_p2_ = {C_1_, D_1_, C_2_, D_2_}.
d) *E*_c00_ = {O_1_O_2_}.
e) *E*_c01_ = {O_1_A_1_, O_1_B_1_, O_2_A_2_, O_2_B_2_}.
f) *E*_c02_ = {A_2_C_1_, A_2_D_1_, B_2_C_2_, B_2_D_2_}.
g) *P*_0_ = {*h*_1_}, where *h*_1_ represents the length of main vein.
h) *P*_1_ = {*l*_1_, *m*_1_, *l*_2_, *m*_2_}, where *l*_1_ and *m*_1_ represent the length of the left and right lateral veins, respectively, at the tail of the bone plate, and *l*_2_ and *m*_2_ represent the length of the left and right lateral veins, respectively, at the head of the bone plate.
i) *P*_2_ = {*u*_1_, *t*_1_, *u*_2_, *t*_2_}, where *u*_1_ and *t*_1_ represent the length of the left and right minor veins, respectively, of the left branch of the bone plate head, and *u*_2_ and *t*_2_ represent the length of the left and right minor veins, respectively, of the right branch of the bone plate head.
j) *P*_3_ = {*α*_1_, *α*_2_}, where *α*_1_ represents the angle between the main vein and the left lateral vein of the head of the T-shaped plate, and *α*_2_ represents the angle between the main vein and the right lateral vein of the head of T-shaped plate.
k) *P*_4_ = {*n*_1_, *d*_1_}, where *n*_1_ represents the thickness of the plate, and *d*_1_ represents the diameter of the screw hole.
l) *F*_00_ = {*X*_p_→*Y*_c_,*| X*_p_ *∈E*_p,_ *Y*_c_ *∈E*_c_}.
m) *F*_01_ = {*Y*_c_→*Z*_s_,*| Y*_c_ *∈E*_c,_ *Z*_s_ *∈E*_s_}.
n) *F*_1_ = {90°<*α*_1_ < 180°, 90°<*α*_2_ < 180°, *u*_1_ = *t*_1_, *u*_2_ = *t*_2_, *l*_1_ = *m*_1_, *l*_1_<*l*_2_<*h*_1_, *d*_1_ < 2*t*_1_ }.

### 2.2 Representation of the bionic vein structure based on digraphs

#### 2.2.1 Method description

**FIGURE 3:**
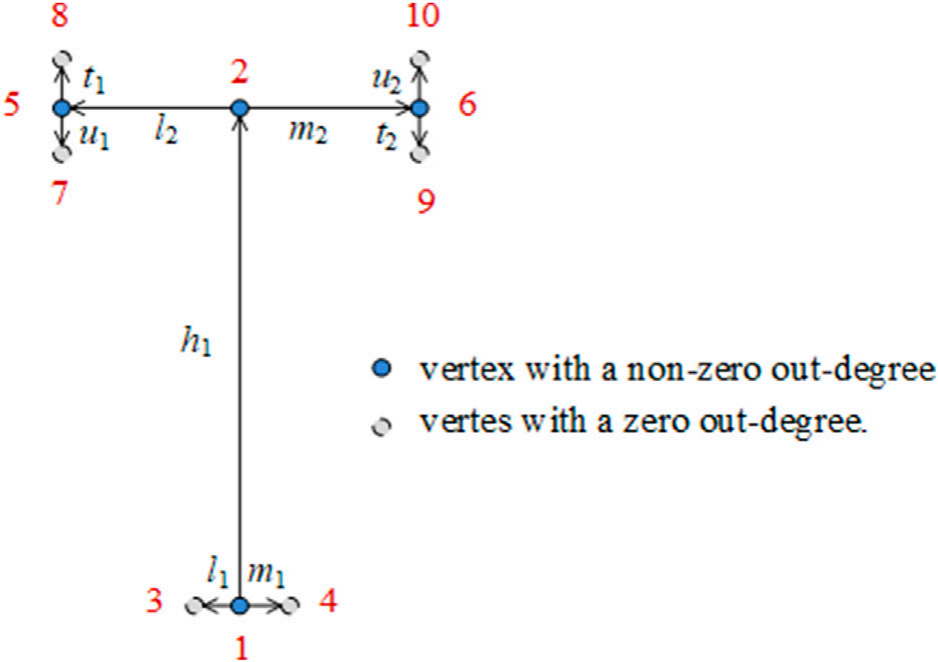
Weighted digraph of the T-shaped plate.

In this study, a weighted digraph was used to represent the bionic vein structure of the implants. A weighted digraph consists of a set of vertices and a set of oriented edges, where the edges are assigned weighted values. Each oriented edge is connected to an ordered pair of vertices. In the weighted digraph, the vertices correspond to the vein feature points and the oriented edges correspond to the vein originating from the main vein feature point to the lateral vein feature point or the vein originating from the lateral vein feature point to the minor feature point. The weighted value corresponds to the length of the vein. As shown in Figure 3, the digraph structure of the implant features is defined as *G* =(*V*, *R*), where *V* represents graph vertices, and *R* represents graph edges. These elements are defined as follows:

a) *V* = {*x*|*x*∈*E*_p_}, where *x* represents the feature points in the bionic vein structure, including the main vein feature points, lateral vein feature points, and minor feature points.

We stipulate the grades of the leaf veins, from high to low, as main vein, lateral vein, and minor vein. We also stipulate that the growth direction of the leaf veins is from high to low. Vertices are numbered from the bottom to the top and from left to right. A directed edge connected to an ordered pair of vertices is represented as <*i*, *j*>. The out-degree of the vertex is also calculated.

**FIGURE 4:**
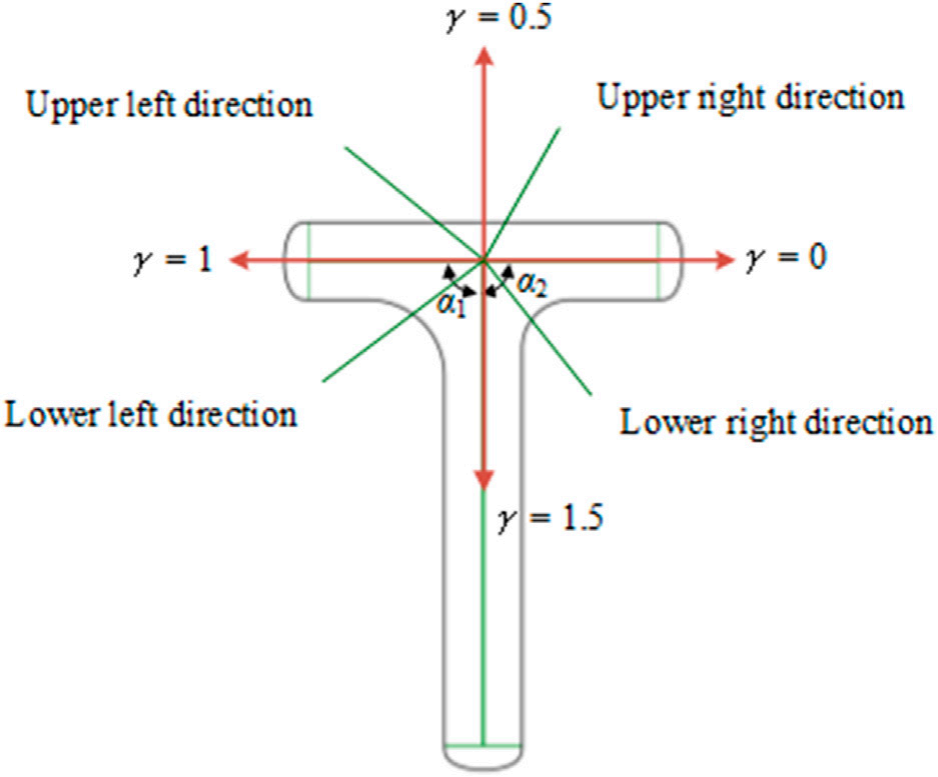
Schematic of the vein direction.

b) *R* = {*e*|*e*∈*E*_c0_}, where *e* represents the leaf veins in the bionic vein structure, including the main veins, lateral veins, and minor veins. *e* contains two properties: *γ* and *v*, which represent the direction angle and length of the vein, separately. We used two adjacency matrices to represent *γ* and *v,* namely the morphological adjacency matrix *M*_1_ and dimensional adjacency matrix *M*_2_, respectively. Considering the growth direction of the veins from a higher level to a lower level, and the number of vertices numbered from a higher level to a lower level, *M*_1_ and *M*_2_ are both upper triangular matrices. As shown in Figure 4, the direction of the lower order vein perpendicular to the main vein and pointing to the right is taken as the reference direction, and *γ* = 0. When rotating counterclockwise, the angle increases by 90°, and *γ* increases by 0.5. The direction of the lower order vein can be determined by *γ* for 0 ≤*γ* < 2, as follows:

0<γ<0.5 Upper right direction

0.5<γ<1 Upper left direction

1<γ<1.5 Lower left direction

1.5<γ<2 Lower right direction

When the direction of the main vein is unchanged, the direction of the lateral vein directly determines the shape of the left and right sides of the T-shaped plate. Combining the two key semantic parameters *α*_1_ and *α*_2_ shown in Figure 2 and incorporating them into *γ* gives the following relation:

$$\gamma =\{\begin{array}{cc} \left(1.5\pi -\alpha_{1}\right)/\pi & 0\leq \alpha_{1}\leq \pi \\ \left(-0.5\pi +\alpha_{2}\right)/\pi & 0.5\pi \leq \alpha_{2}\leq \pi \\ \left(1.5\pi +\alpha_{2}\right)/\pi & 0\leq \alpha_{2}<0.5\pi \end{array}$$ (1)

The adjacency matrices *M*_1_ and *M*_2_ of the bionic vein structure of the T-shaped bone plate are as follows:

$$M_{1ij}=\{\begin{array}{cc} \gamma_{ij}, & <i,j>\in E_{p}\\ 0, & \mathrm{Other}\;\mathrm{situations} \end{array}$$ (2)

$$M_{2ij}=\{\begin{array}{cc} v_{ij}, & <i,j>\in E_{p}\\ 0, & \mathrm{Other}\;\mathrm{situations} \end{array}$$ (3)

**FIGURE 5:**
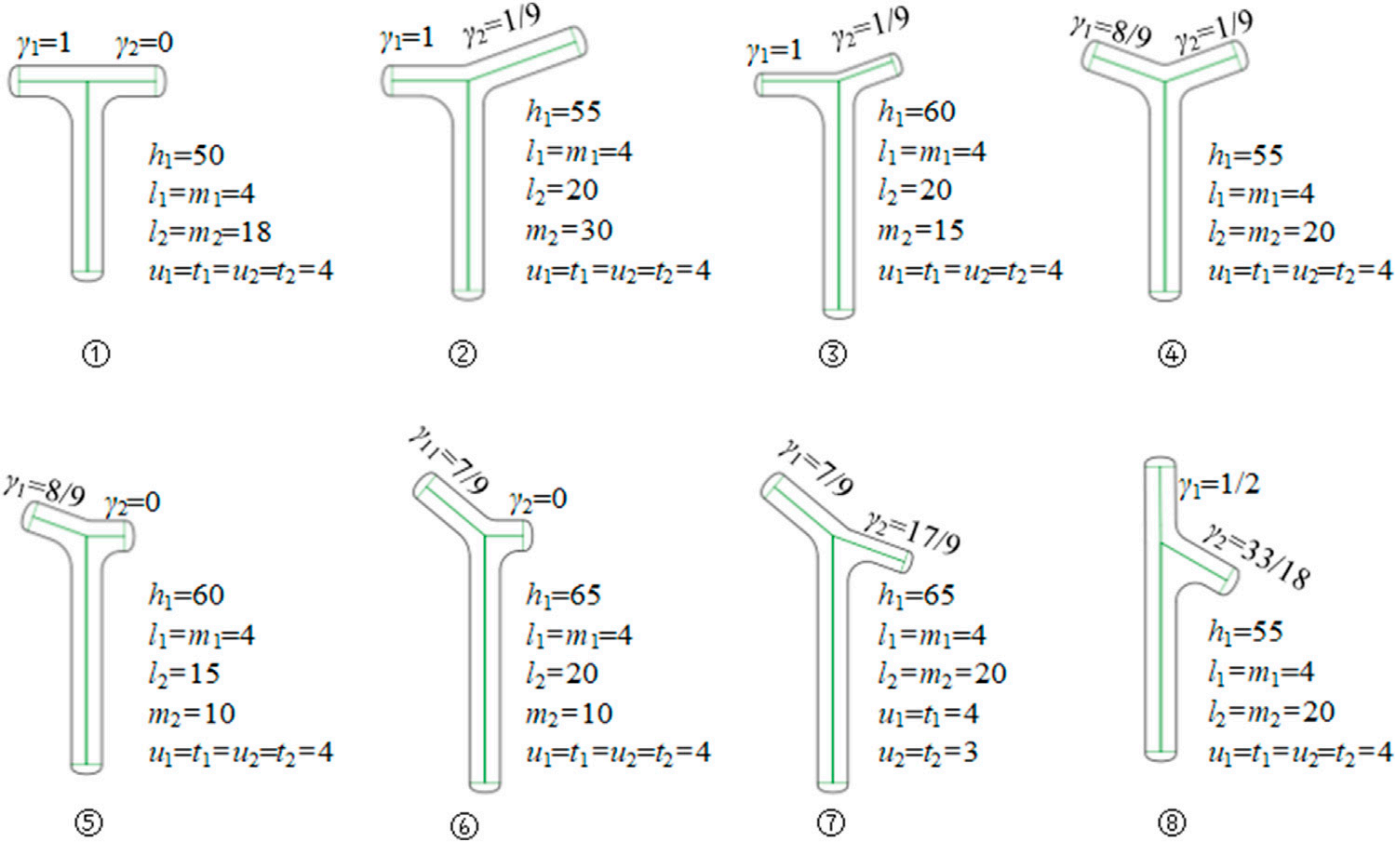
Bionic vein structure features of customized T-shaped plates. By modifying semantic parameters in Figure 2B, the adjacency matrices *M_1_* and *M_2_* are updated, and then different bionic vein structure features are generated

By modifying the adjacency matrices *M*_1_ and *M*_2_, the bionic vein structure features representing different shapes and sizes are generated. They can subsequently be added to the feature database to facilitate retrieval later. Figure 5 shows the bionic vein structure feature of a partially T-shaped bone plate.

#### 2.2.2 Example analysis

Bionic vein structures have been widely used in the fabrication of a variety of implants. Based on the function of the bionic vein structure on the implant model design, implants can be divided into overall structure representations and interest region structure representations. The overall structure representation is generally used for implants with relatively simple shapes such as the T-shaped plate and the L-shaped plate. The representation of the interest region structure is suitable for complex implants, such as the head of the clover bone plate, and the trochanter of the femoral stem.

##### 2.2.2.1 L-shaped plate

**FIGURE 6:**
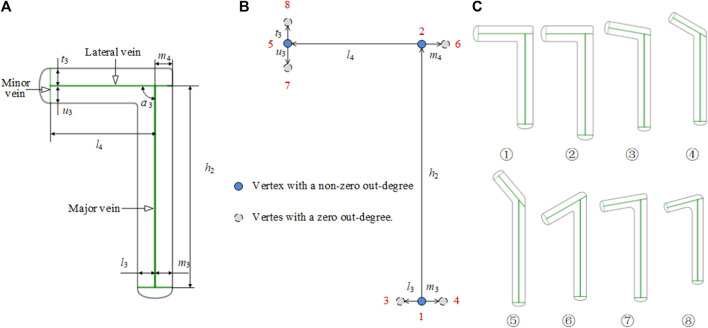
Bionic vein structure features of the L-shaped plate. **(A)** Semantic parameters of L-shaped bone plate, mainly refers to the length of veins and the angle between veins. **(B)** Weighted digraph of L-shaped bone plate. Vertices are numbered from the bottom to the top and from left to right. The morphological and dimensional adjacency matrices corresponding to the digraph are *M_3_* and *M_4_* respectively **(C)** Bionic vein structure features of customized L-shaped plates. By modifying semantic parameters in **(A)**, *M_3_* and *M_4_* are updated, and then different bionic vein structure features are generated.

An L-shaped plate is divided into a left L-shaped plate and a right L-shaped plate. As shown in Figure 6, taking the left L-shaped bone plate as an example, the tail center line is selected as the main vein, and the lower end is branched out to form the lateral veins that define the width of the tail. At the head, the central line of the left branch serves as the lateral vein, and the right vein is in the same line as the left one in the opposite direction. The left vein branches off into the left and right minor veins that are used to define the width of the left branch. The L-shaped bone plate has a bionic vein structure and morphology adjacency matrices *M*_3_ and *M*_4_. By adjusting *M*_3_ and *M*_4_, different shapes and sizes of the L-shaped bone plate can be generated. *M*_3_ and *M*_4_ are as follows:

$$M_{3}=\left[\begin{array}{cccccccc} 0 & 0.5 & 1 & 0 & 0 & 0 & 0 & 0\\ & 0 & 0 & 0 & \gamma_{3} & 1.5-\gamma_{3} & 0 & 0\\ & & 0 & 0 & 0 & 0 & 0 & 0\\ & & & 0 & 0 & 0 & 0 & 0\\ & & & & 0 & 0 & 1.5 & 0.5\\ & & & & & 0 & 0 & 0\\ & & & & & & 0 & 0\\ & & & & & & & 0 \end{array}\right]$$

$$M_{4}=\left[\begin{array}{cccccccc} 0 & h_{2} & l_{3} & m_{3} & 0 & 0 & 0 & 0\\ & 0 & 0 & 0 & l_{4} & m_{4} & 0 & 0\\ & & 0 & 0 & 0 & 0 & 0 & 0\\ & & & 0 & 0 & 0 & 0 & 0\\ & & & & 0 & 0 & u_{3} & t_{3}\\ & & & & & 0 & 0 & 0\\ & & & & & & 0 & 0\\ & & & & & & & 0 \end{array}\right]$$

##### 2.2.2.2 Clover bone plate

**FIGURE 7:**
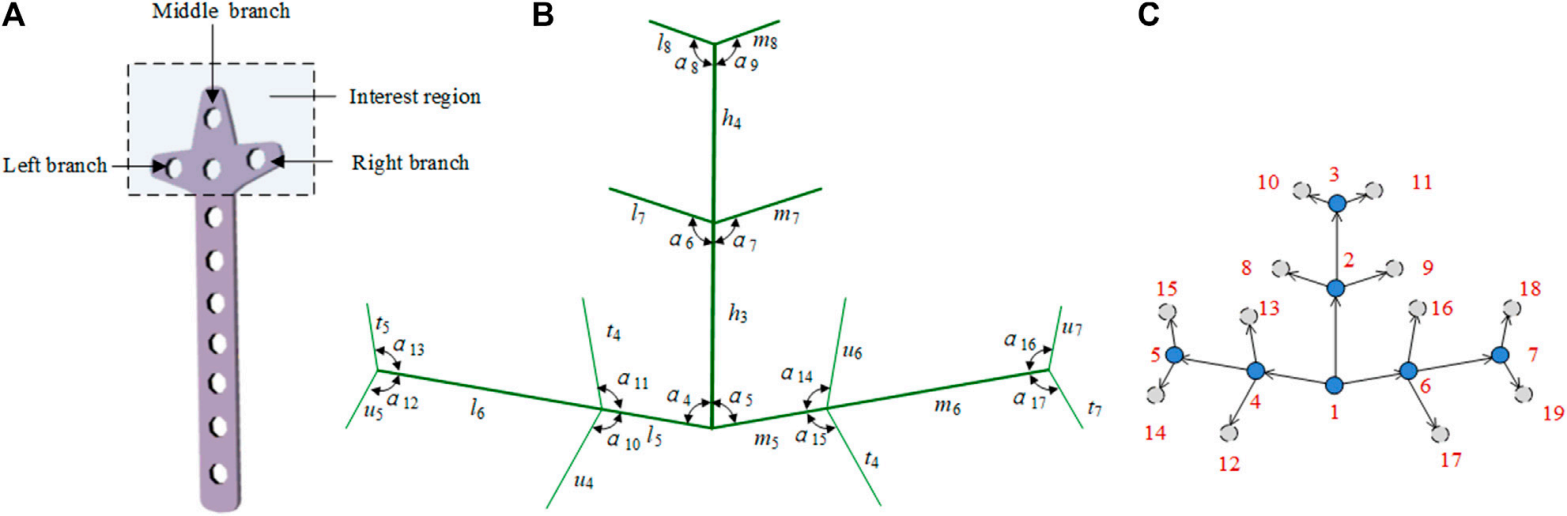
Bionic vein structure feature of the clover bone plate. **(A)** Interest region of the clover bone plate, contains three branches: left branch, middle branch, and right branch. **(B)** Semantic parameters of the interest region, mainly refers to the length of veins and the angle between veins. **(C)** Weighted digraph of the interest region.

In a clover bone plate, the design of the three branches of the head structure is very important. To improve the design efficiency of the head structure, we constructed the head of the clover bone plate with a bionic vein structure. As shown in Figure 7A, the interest region is divided into the left branch, middle branch, and right branch. As shown in Figure 7B, the center line of the middle branch is considered the main vein. At the lower end of the main vein, the center line of the left branch is taken as the left lateral vein, and the center line of the right branch is taken as the right lateral vein. Two left lateral veins and two right lateral veins are added at the center and the top of the main vein to facilitate the adjustment of the middle branch. Similarly, two minor veins are added to each side of the lateral veins. The 32 semantic parameters set in Figure 7B are used to construct their adjacency matrices. And the digraph is shown in Figure 7C.

##### 2.2.2.3 Femoral stem prosthesis

**FIGURE 8:**
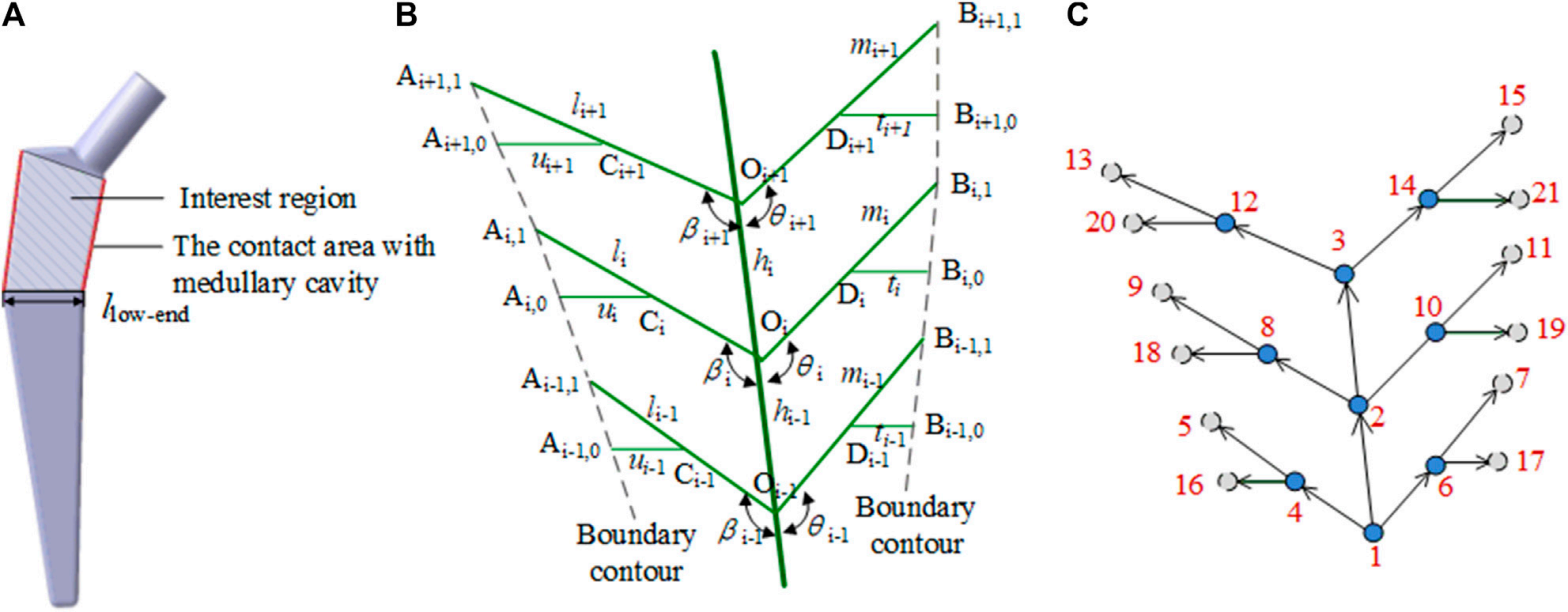
Bionic vein structure feature of the femoral stem prosthesis. **(A)** Interest region of the femoral stem prosthesis. **(B)** Semantic parameters of the interest region, mainly refers to the length of veins and the angle between veins. **(C)** Weighted digraph of the interest region.

Bionic vein structures can also be used to design the interest region of the implants, such as the femoral stem, as shown in Figure 8A. To construct a femur stem prosthesis that is a good match for the individual patient, modifications in this area are critical. The bionic vein structure can make local adjustments to the interest region more convenient.

As shown in Figure 8B, the center line O_i−1_O_i+1_ of the interest region is used as the main vein. The left side branches out into the left lateral vein O_i_A_i,1_. The left lateral vein further branches out into the left minor vein C_i_A_i,0_, where C_i_ is the midpoint of O_i_A_i,1_. The right side branches out into the right lateral vein O_i_B_i,1_. The right lateral side branches out into the right minor vein D_i_B_i,0_, where D_i_ is the midpoint of O_i_B_i,1_. As shown in Figure 8C, 18 parameters, such as vein length and angle between the veins, are set and their adjacency matrices can be constructed according to these parameters.

### 2.3 Bionic vein structure feature retrieval method

**FIGURE 9:**
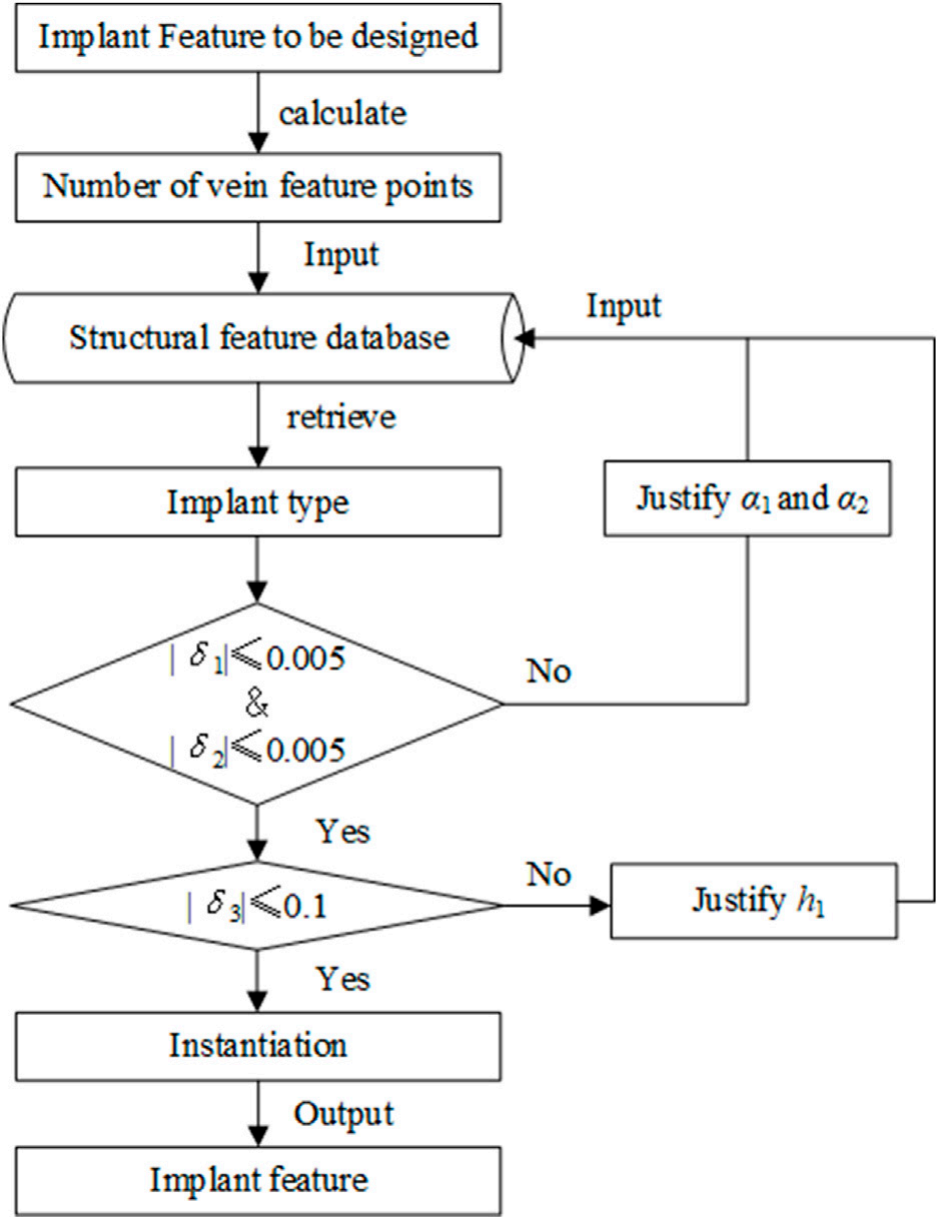
Bionic vein structure retrieval method.

The purpose of retrieval is to find the most consistent feature of an implant from the bionic vein structure feature database. To improve retrieval efficiency, as shown in Figure 9, we adopted a method in which the shape is first matched and then the size is modified. The specific steps are as follows:

Step 1: Search and determine the required implant types according to the number of vein feature points. The number of vein feature points determines the size of the morphological adjacency matrix. For example, the T-shaped bone plate has 10 vein feature points, and its morphological adjacency matrix is 10×10.  Taking the T-shaped bone plate as an example, further retrieval is as follows:
Step 2: Determine the overall shape of the T-shaped bone plate using the key elements in the morphological adjacency matrix. Calculate the errors of *γ*_1_ and *γ*_2_ with the corresponding expected values as *δ*_1_ and *δ*_2_, respectively. If $\left|\delta_{1}\right|\leq 0.005\;\mathrm{and}\;\left|\delta_{2}\right|\leq 0.005$ , then *α*_1_ and *α*_2_ error values lie within one degree, and the implant shape can be directly selected for the structure feature. If the conditions are not met, *α*_1_ and *α*_2_ must be adjusted to generate the desired features and inserted into the feature database.
Step 3: Determine the implant size based on the dimensional adjacency matrix;
  Step 3.1: In step 2, the structural features with a maximum matching degree were selected. An error value defined by *δ*_3_ is calculated by comparing the length of main vein *h*_1_ in the adjacency matrix. If $\left|\delta_{3}\right|\leq 0.1$ , the T-shaped plate size roughly conforms to the requirements of the bone and can be used as the feature structure. If the condition is not met, *h*_1_ can be adjusted.
  Step 3.2: Based on the instantiation method, the required dimensions are generated by adjusting the remaining semantic parameters and adding them to the feature database.

## 3 Results and discussion

The clover bone plate and the femoral stem prosthesis were tested to explain and analyze our method. The hardware configuration used for our experiment includes an Intel(R) Core(TM) i7-9750H CPU at 2.60 and 8 GB RAM, and the software used were Visual C++ and the geometric modeling engine CATIA P3 V5R21.

### 3.1 Clover bone plate

#### 3.1.1 Entity model construction

**FIGURE 10:**
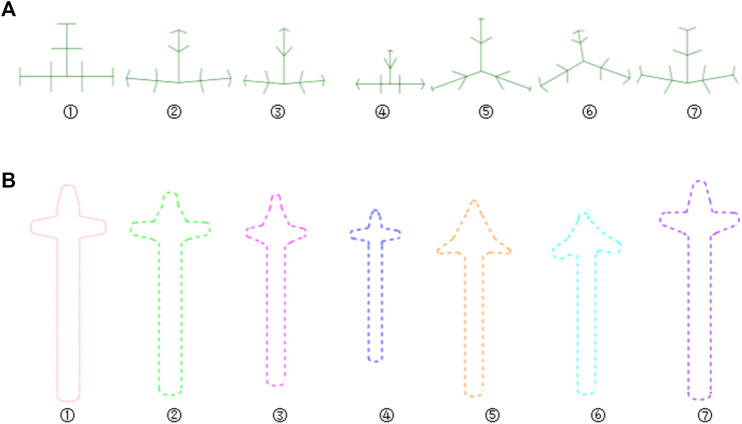
Bionic vein structures and sketches of clover bone plate. **(A)** Bionic vein structures generated by setting different semantic parameters shown in Table 1. These structures are numbered 1 through 7. **(B)** Feature sketches of the structures in panel **(A)**.

The feature database of bionic vein structure was built based on parameters in Table.1, and their bionic vein structures and their sketch are shown in Figure 10.

**TABLE 1:** Parameters of the interest region (Unit of length: mm; Unit of Angle:°).

No.	*α*_4_	*α*_5_	*α*_6_	*α*_7_	*α*_8_	*α*_9_	*α*_10_	*α*_11_	*α*_12_	*α*_13_	*α*_14_	*α*_15_	*α*_16_	*α*_17_	*h*_3_	*h*_4_
	*l*_5_	*l*_6_	*m*_5_	*m*_6_	*l*_7_	*m*_7_	*l*_8_	*m*_8_	*u*_4_	*t*_4_	*u*_5_	*t*_5_	*u*_6_	*t*_6_	*u*_7_	*t*_7_
1	90	90	90	90	90	90	90	90	90	90	90	90	90	90	9	8
	5	10	5	10	5	5	3	3	5	5	3	3	5	5	3	3
2	85	85	120	120	70	70	100	100	70	70	100	100	70	70	10	7
	7	10	7	10	4	4	3	3	4	4	3	3	4	4	3	3
3	85	85	140	140	60	60	110	110	70	70	110	110	70	70	9	9
	5	8	5	8	4	2	4	2	4	2	4	2	4	2	4	2
4	90	90	150	150	60	60	90	90	60	60	90	90	60	60	5	6
	3	8	3	8	4	4	1	1	3	3	2	2	3	3	2	2
5	110	110	120	120	70	70	140	140	70	70	140	140	70	70	9	8
	5	12	5	12	4	4	1	1	5	5	1	1	5	5	1	1
6	110	120	120	120	70	80	120	120	70	70	110	110	60	60	6	4
	6	10	6	10	3	3	2	2	4	4	3	3	4	4	3	3
7	80	80	110	110	110	110	110	110	110	110	110	110	110	110	9	8
	5	5	3	3	5	5	3	3	5	5	3	3	5	5	3	3

**FIGURE 11:**
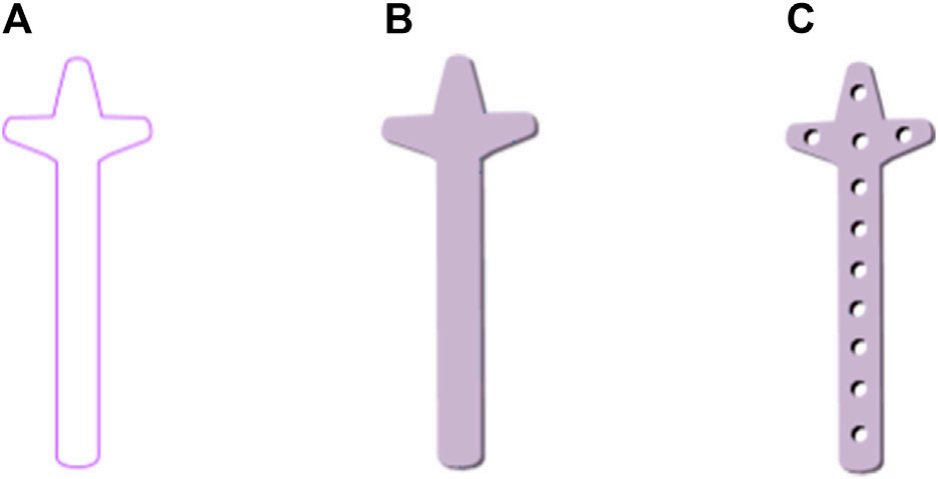
Clover bone plate generation based on bionic vein structure. **(A)** Feature sketch No.7 in Figure 10B. **(B)** Entity model is generated by filling the feature sketch in panel **(A)** to generate surface, and then stretching the surface. **(C)** Clover bone plate is generated by punching screw holes based on the entity model shown in panel **(B)**.

The design procedure consists of the following steps. First, the feature sketch was retrieved from the feature database, taking the matrix as the index key; and then the entity model was rapidly built by stretching the sketch and adding dress-up feature. The entity model of sketch No.7 is shown in Figure 11.

#### 3.1.2 Fit Degree Calculation

Plate designs with high levels of anatomical compliance are demonstrated to have numerous clinical benefits (Andreas et al., 2018; Schmutz et al., 2019). To measure the fit degree between clover bone plate and bone surface, Hausdorff distance (Langerak, 2010; van Kreveld et al., 2022) has been proven to perform well for the assessment of surface similarity is used. Vein feature points of clover bone plate and corresponding points on the bone surface are separately selected as point sets to evaluate the fit degree. If the Hausdorff distance between the abutted surface and bone surface is less than 0.3 mm, then the fit degree is valid. In this study, the distance is 0.17 mm which indicates a good fit degree.

In addition, the fit degree calculation (Liu et al., 2020; Zhu et al., 2021) is also referred. When the average distance between the bone plate and bone is less than 2 mm, the corresponding fitting index is greater than 0.5, and the fitting property of the bone plate is excellent. In this study, the result is 0.682, which indicates a fitting property.

#### 3.1.3 Mechanical property analysis

**FIGURE 12:**
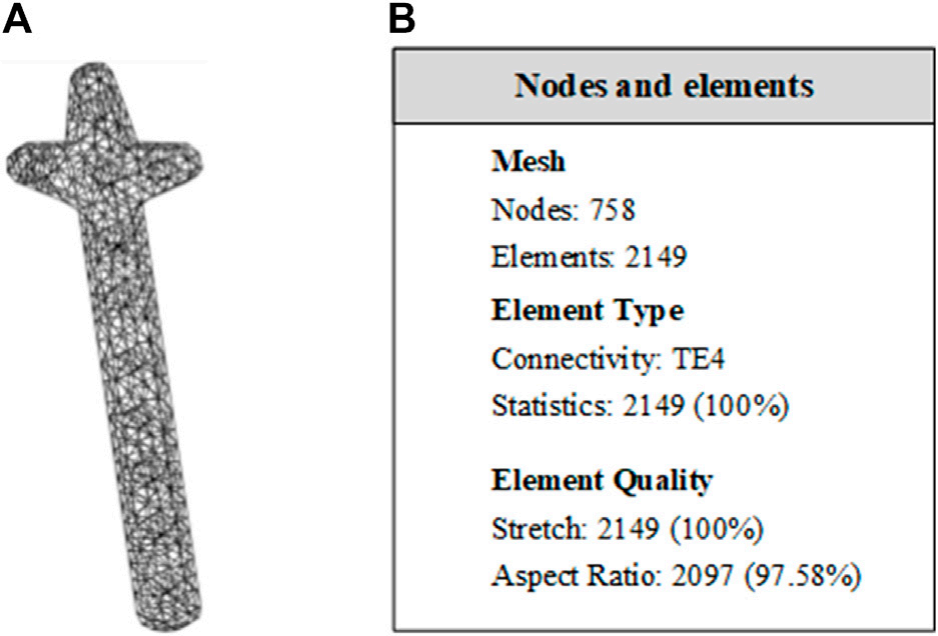
Entity meshing model of clover bone plate. **(A)** The clover bone plate after meshing. In grid division, the absolute sag was set to 2 mm, and mesh size was set to 10 mm. **(B)** Nodes and elements of grid division. The aspect ratio was 97.58% and the streth 100%, showing the meshing result is good.

**FIGURE 13:**
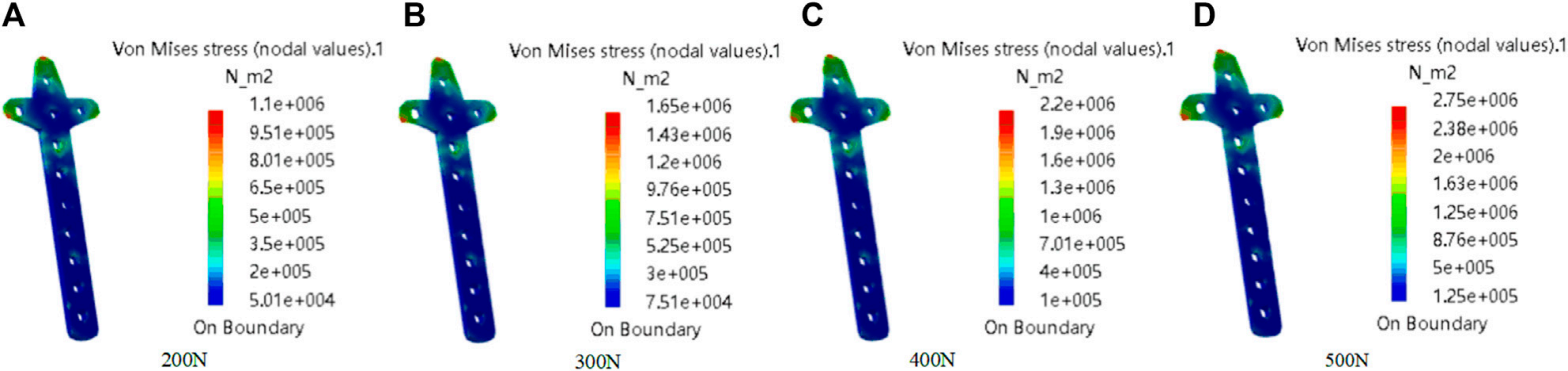
Mechanical property analysis of clover bone plate. The distributed force was applied to the upper of the model. From **(A–D)**, the force values are 200N, 300N, 400N, and 500N.

In this study, the von Mises Stress of the clover bone plate was studied by meshing, additional materials, adding constraints and loads, and calculating results, to verify the good mechanical properties of the clover bone plate. Titanium alloy was added to the entity model, its elastic modulus, yield, and tensile strength were 1.14 × 10^11^ Pa and 8.25 × 10^8^ Pa, respectively. In grid division, the absolute sag was set to 2 mm, and mesh size was set to 10 mm. The results of grid division showed that the aspect ratio was 97.58% and the stretch 100%, showing that the mesh-griding was good, as shown in Figure 12. The distributed force of 200, 300, 400, and 500 N was applied to the upper surface of the model. The experiment showed that the stress distribution was relatively balanced, as shown in Figure 13. The above experiments show that the designed models had better mechanical properties and can meet the clinic’s needs.

### 3.2 Femoral stem prosthesis

The handle region of the femoral stem fits into the femur cavity, its stability was important to the treatment of femoral head necrosis. Therefore, the handle region of the femoral stem is taken as the experimental case.

#### 3.2.1 Entity model construction

**FIGURE 14:**
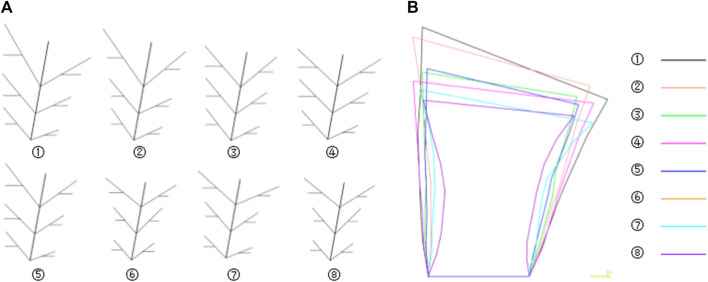
Bionic vein structures and sketches of femoral stem prosthesis. **(A)** Bionic vein structures generated by setting different semantic parameters shown in Table 2. These structures are numbered 1 through 7. **(B)** Feature sketches of the structures in panel **(A)**.

The feature database of bionic vein structure has been built based on parameters shown in Table 2, and their bionic vein structures and sketch are shown in Figure 14.

**TABLE 2:** Parameters of the interest region (Unit of length: mm; Unit of Angle:°).

Number	*β_1_*	*β_2_*	*β_3_*	*θ_1_*	*θ_2_*	*θ_3_*	*l_1_*	*l_2_*	*l_3_*	*m_1_*	*m_2_*	*m_3_*	*u_1_*	*u_2_*	*u_3_*	*t_1_*	*t_2_*	*t_3_*
1	130	130	140	120	125	130	27	33	46	19	25	37	8	11	12	7.5	9	13
2	135	130	135	125	125	140	25	29	45	20	23	35	7	8	10	7.5	8.5	9.5
3	132	130	129	127	135	140	26	30	35	20	24	30	8	9	11	7.5	9	9
4	128	125	123	125	130	132	25	30	35	21	26	33	8	11	12	7.5	9	10
5	130	125	132	120	133	136	24	27	35	17	22	29	8	10	11	7.5	8	9
6	126	123	126	126	137	139	20	22	29	18	23	26	7	9	9	7	7	8
7	126	129	122	122	135	123	20	25	32	18	21	30	7	8	9	7	7	8
8	129	129	119	137	145	132	17	22	29	18	23	26	7	6	9	7	6	6

**FIGURE 15:**
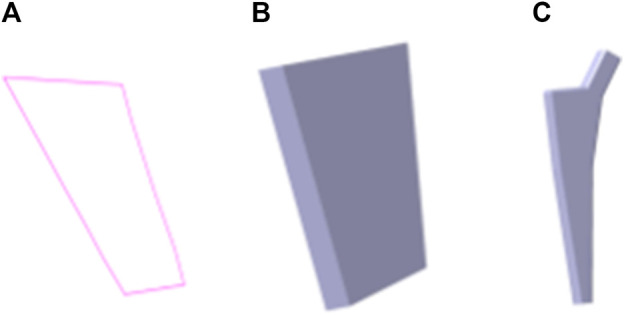
Femoral stem prosthesis generation based on bionic vein structure. **(A)** Feature sketch No.7 in Figure 14B. **(B)** Entity model is generated by filling the feature sketch in panel A to generate surface, and then stretching the surface. **(C)** Clover bone plate is generated based on the entity model shown in panel **(B)**.

The design procedure consists of the following steps. First, the feature sketch was retrieved from the feature database, taking the matrix as the index key; and then the entity model was rapidly built by stretching the sketch and adding other entity portions. The entity model of sketch No.7 is shown in Figure 15.

#### 3.2.2 Mechanical property analysis

**FIGURE 16:**
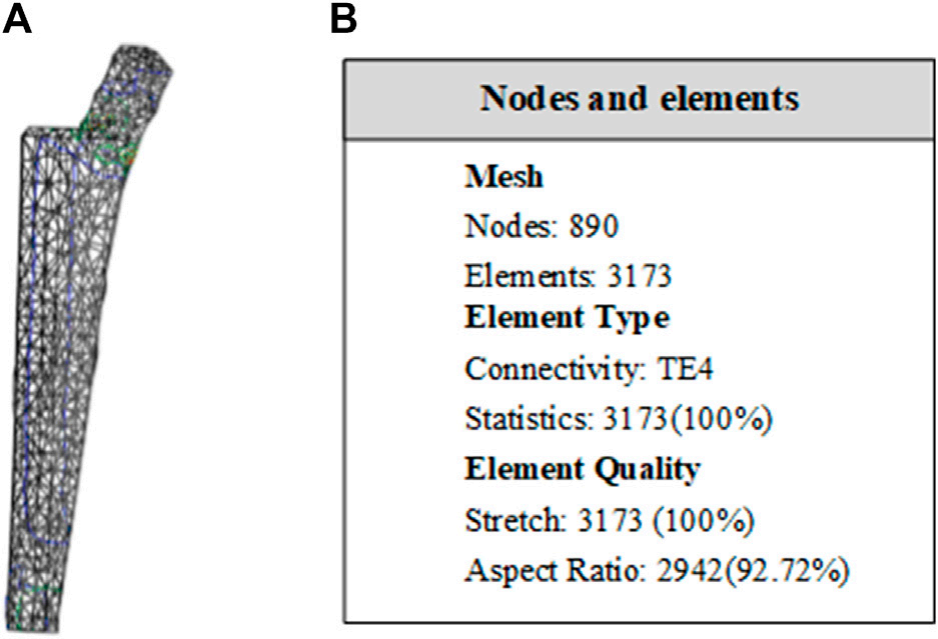
Entity meshing model of femoral stem. **(A)** The model after meshing. In grid division, the absolute sag was set to 2 mm, and mesh size was set to 10 mm. **(B)** Nodes and elements of grid division. The aspect ratio was 92.72% and the streth 100%, showing the meshing result is good.

**FIGURE 17:**
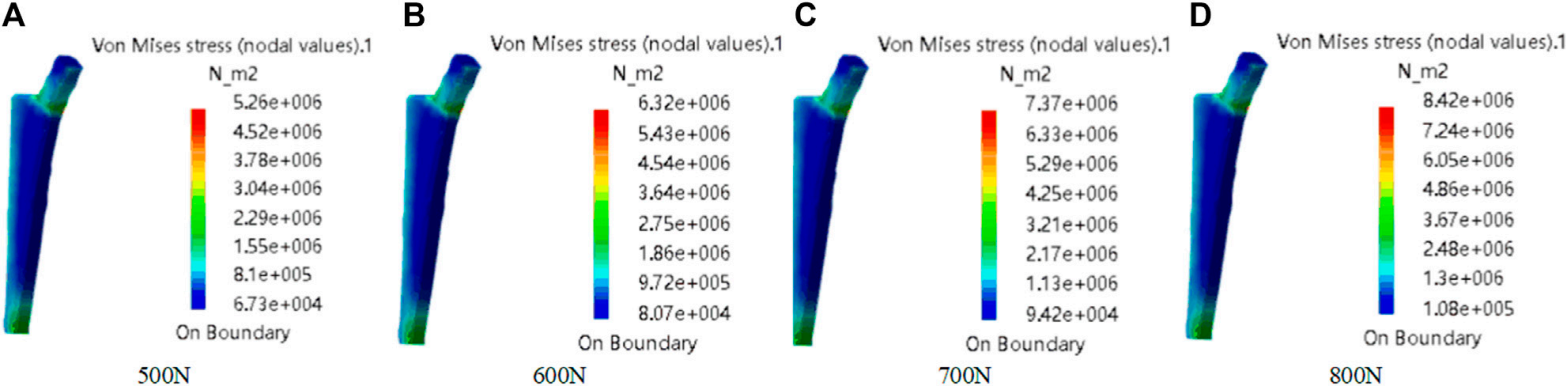
Mechanical property of femoral stem prosthesis. The distributed force was applied to the proximal end of model. From **(A–D)**, the force values are 500N, 600N, 700N, and 800N.

The material is the same as the clover plate, but its properties are slightly different. Its elastic yield and tensile strengths are 1.14 × 10^11^ Pa and 8.25 × 10^8^ Pa, respectively. In grid division, the absolute sag was set to 2 mm, and the size was set to 10 mm. The result of grid division shows that the aspect ratio was 92.72% and the stretch 100%, showing that the mesh-griding is good, as shown in Figure 16. The distributed force of 500, 600, 700, and 800 N was applied to the proximal end of the model based on the clinic experience. The experiment shows that the stress distribution was relatively balanced, as shown in Figure 17, and was able to meet the clinic's needs.

The customized bone plate was constructed by using the adjacent matrix based on the bionic vein structure. And the adjacency matrix integrated the morphological and dimensional features, thus achieving efficient modification and redesign. The comparison with the existing methods was shown in Table 3. The result shows that our method is simple and efficient.

**TABLE 3:** Comparison between the proposed method and existing methods.

Compare item	Soni (Soni and Singh (2020)	Babaniamansour (Babaniamansour et al. (2017)	Liu (Liu et al. (2019)	Chen (Chen et al. (2021)	He (He et al. (2019)	Wang (Wang et al. (2017)	This method
Parameterized?	No	Yes	No	Yes	Yes	Yes	Yes
Integrated?	No	No	No	No	No	No	Yes
Convenient feature retrieval?	—	—	—	—	—	—	Yes
Is fit degree satisfactory?	Yes	Yes	Yes	Yes	Yes	Yes	Yes
Is mechanical property satisfactory?	Yes	Yes	—	—	—	Yes	Yes
Operability	General	General	Simple	Simple	Simple	Simple	Simpler
Redesign	Complex	Complex	General	Simple	Simple	Simple	Simpler

## 4 Conclusion

Customized implant design was a persistently relevant research hotspot. In the current research, the relevant information was fragmented, which hinder the customized implant design. In order to improve the personalized design efficiency, the bionic vein structure feature was proposed. The main contributions of this study were as follows.

1) The implant abutted surface was first represented by a bionic vein structure, which contained vein feature points, vein feature curves, and semantic parameters.
2) The bionic vein structure presentation was represented as a weighted digraph, including the morphological and dimensional adjacency matrices, which were added to the feature database of bionic vein structure to facilitate the implant retrieval.
3) The design of the customized implant was achieved efficiently by retrieving the relevant matrix from the feature database and instancing the parameters of the bionic vein structure.

The experimental results showed that the proposed method was simple, flexible, and efficient, and can quickly design the implant with better mechanical properties. The proposed method can provide innovative ideas, methods, and techniques for other fields, such as mechanical design and virtual scene construction, but there are many research details for the implementation in other fields, which was our further work.

## Data Availability

The original contributions presented in the study are included in the article/Supplementary Material, further inquiries can be directed to the corresponding author.
