# Supplementary figures and images for: Convenient design method for customized implants based on bionic vein structure features
Source: Front Bioeng Biotechnol. 2022 Aug 12;10:929133. doi: 10.3389/fbioe.2022.929133 (PMC9412103; doi:10.3389/fbioe.2022.929133)

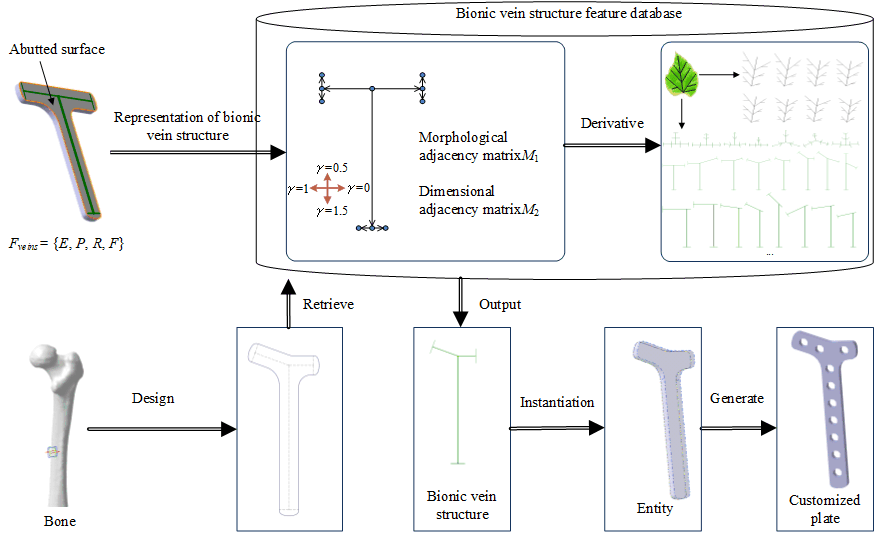

Supplement: Supplementary file 1 [file DataSheet1.zip › Figures/1.png]

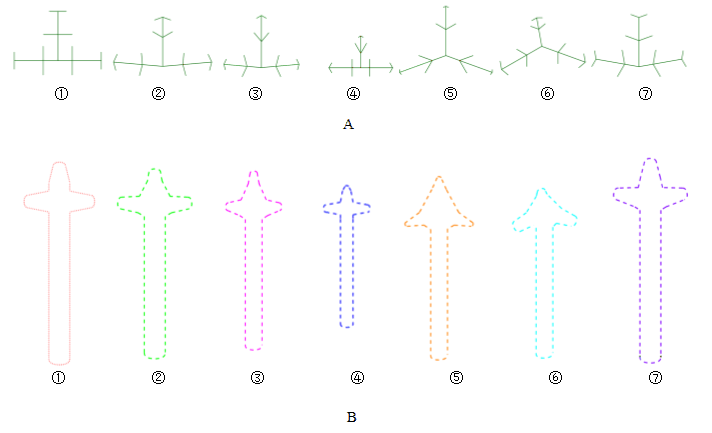

Supplement: Supplementary file 1 [file DataSheet1.zip › Figures/10.png]

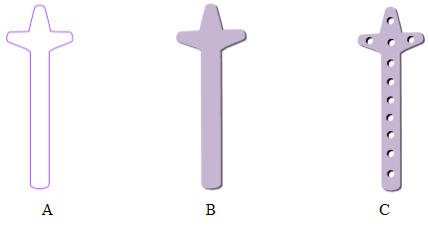

Supplement: Supplementary file 1 [file DataSheet1.zip › Figures/11.png]

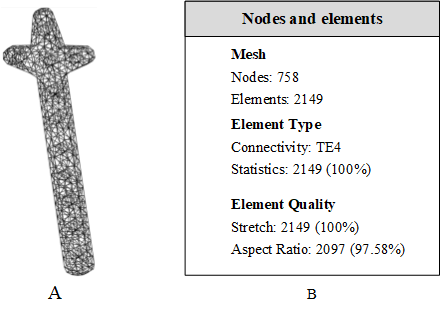

Supplement: Supplementary file 1 [file DataSheet1.zip › Figures/12.png]

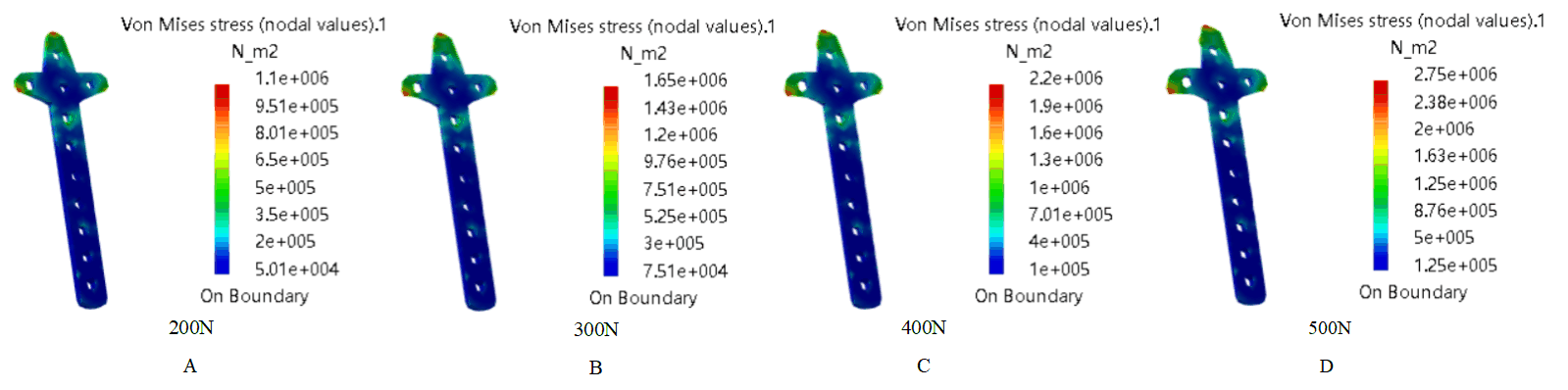

Supplement: Supplementary file 1 [file DataSheet1.zip › Figures/13.png]

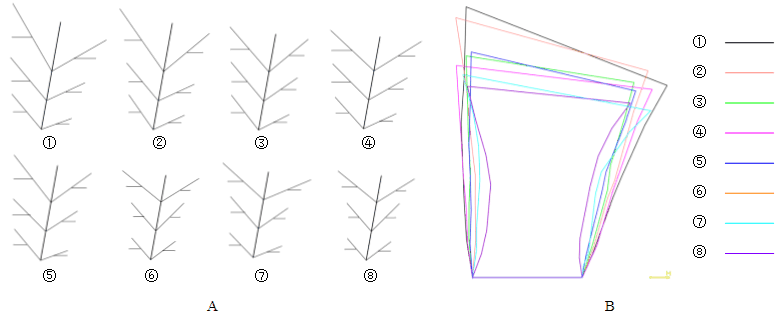

Supplement: Supplementary file 1 [file DataSheet1.zip › Figures/14.png]

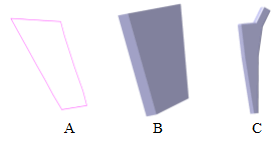

Supplement: Supplementary file 1 [file DataSheet1.zip › Figures/15.png]

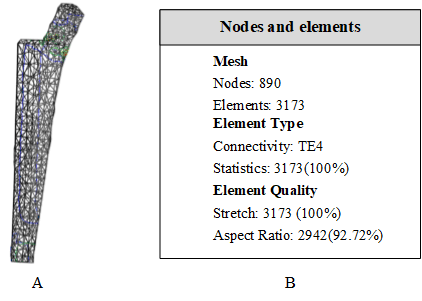

Supplement: Supplementary file 1 [file DataSheet1.zip › Figures/16.png]

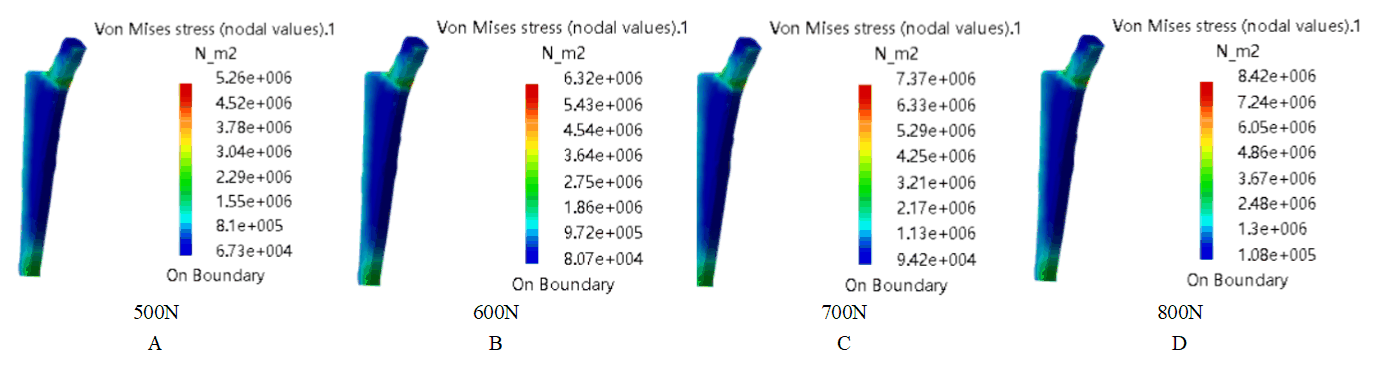

Supplement: Supplementary file 1 [file DataSheet1.zip › Figures/17.png]

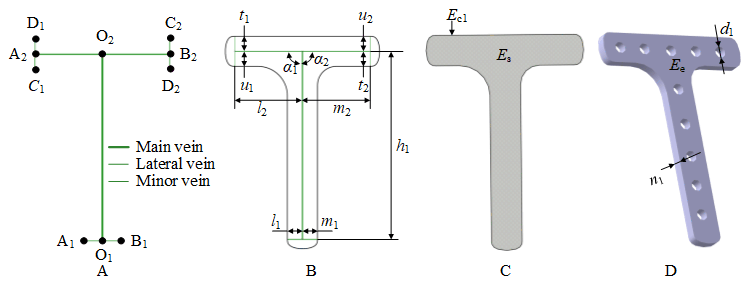

Supplement: Supplementary file 1 [file DataSheet1.zip › Figures/2.png]

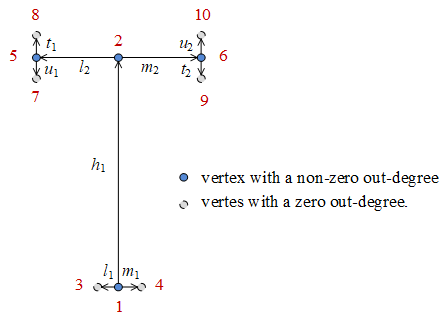

Supplement: Supplementary file 1 [file DataSheet1.zip › Figures/3.png]

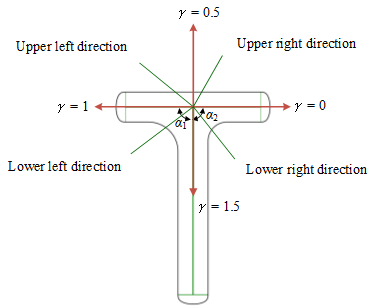

Supplement: Supplementary file 1 [file DataSheet1.zip › Figures/4.png]

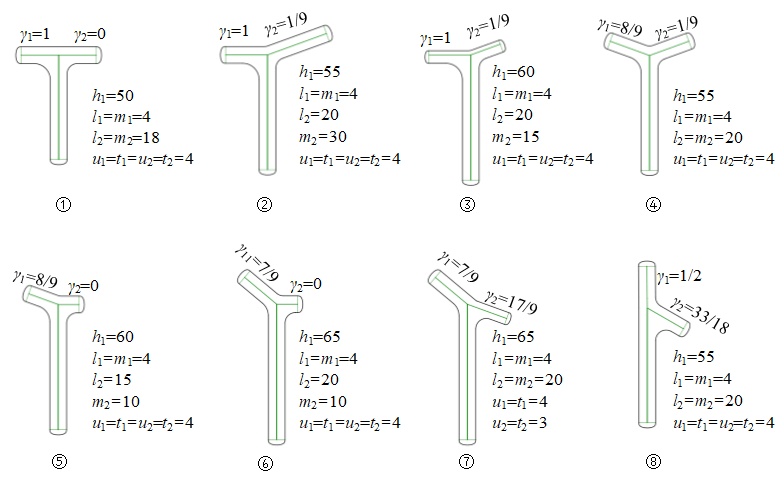

Supplement: Supplementary file 1 [file DataSheet1.zip › Figures/5.png]

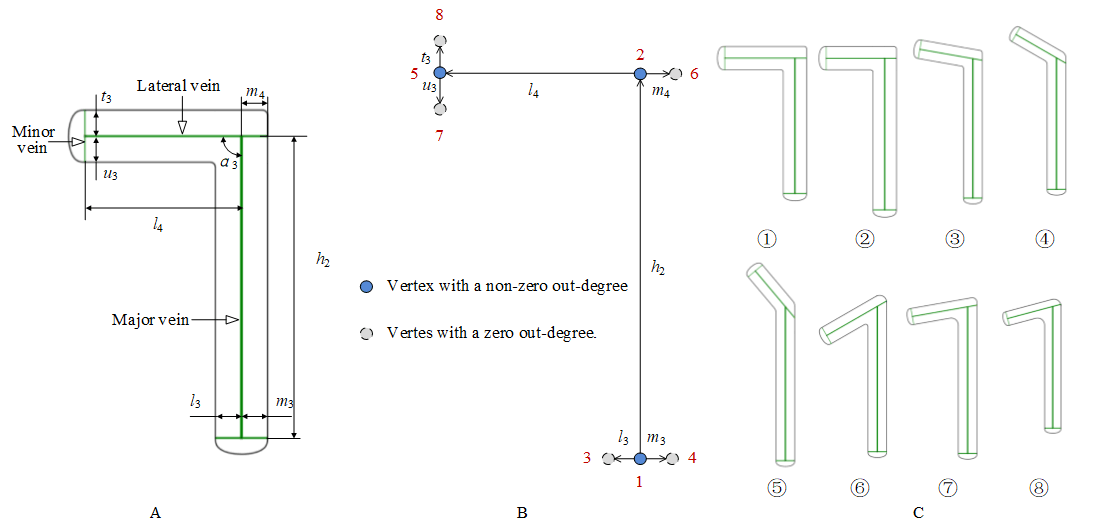

Supplement: Supplementary file 1 [file DataSheet1.zip › Figures/6.png]

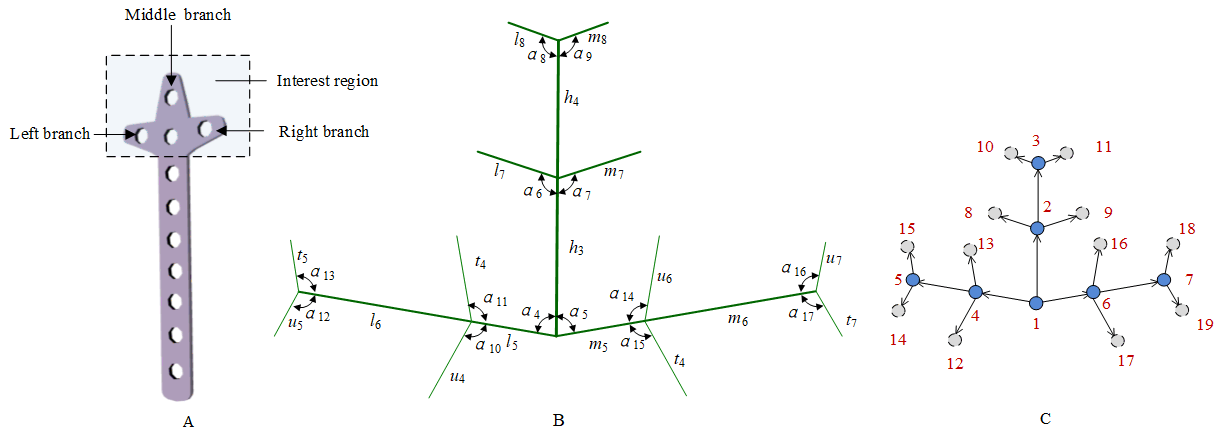

Supplement: Supplementary file 1 [file DataSheet1.zip › Figures/7.png]

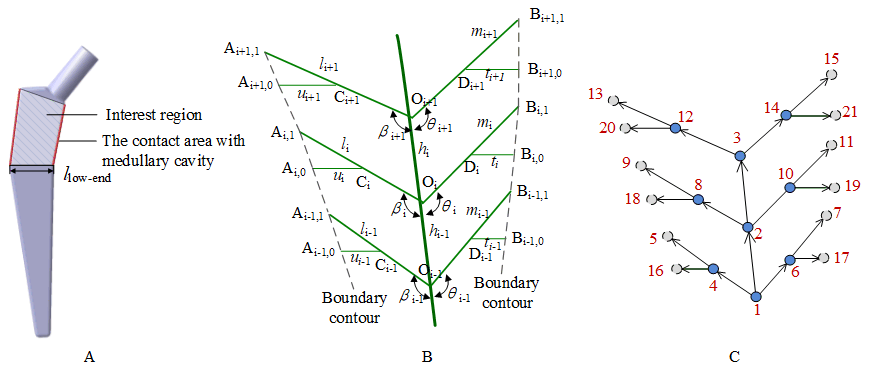

Supplement: Supplementary file 1 [file DataSheet1.zip › Figures/8.png]

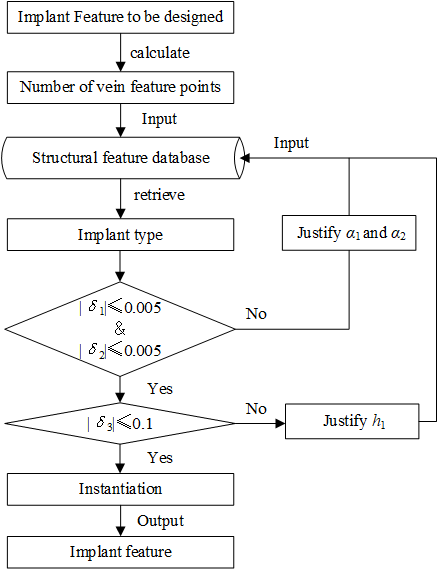

Supplement: Supplementary file 1 [file DataSheet1.zip › Figures/9.png]
